# Long-term outcomes of active vaccination against de novo hepatitis B among pediatric recipients of living donor liver transplantations with anti-HBc (+) grafts: a retrospective case–control study

**DOI:** 10.1097/JS9.0000000000001801

**Published:** 2024-06-13

**Authors:** Chee-Chien Yong, Yu-Hung Lin, Wendell Z. Espinosa, I-Hsuan Chen, Shih-Ho Wang, Yi-Chia Chan, Chao-Long Chen, Chih-Che Lin

**Affiliations:** a Liver Transplantation Center and Department of Surgery, Kaohsiung Chang Gung Memorial Hospital, Taiwan; b Chang Gung University College of Medicine, Taiwan; c Kaohsiung Municipal Feng Shan Hospital-Under the management of Chang Gung Medical Foundation, Kaohsiung, Taiwan; d Department of Internal Medicine, College of Medicine, University of St. La Salle, Philippines

**Keywords:** core antibody, de novo, HBV, liver transplantation

## Abstract

**Background:**

Active vaccination has been utilized to prevent de novo hepatitis B virus infection (DNHB) in anti-HBc (+) grafts after liver transplantation. However, the long-term efficacy of active vaccination and graft/patient outcomes of anti-HBc (+) grafts have yet to be comprehensively investigated.

**Materials and methods:**

Among 204 pediatric patients enrolled in the study, 82 recipients received anti-HBc (+) grafts. For DNHB prevention, active vaccination was repeatedly administered prior to transplant. Antiviral therapy was given to patients with pretransplant anti-HBs <1000 IU/ml (nonrobust response) for 2 years and discontinued when post-transplant patients achieved anti-HBs >1000 IU/ml, while antiviral therapy was not given in patients with an anti-HBs titer over 1000 IU/ml. The primary outcome was to investigate the long-term efficacy of active vaccination, while the secondary outcomes included the graft and patient survival rates.

**Results:**

Among the 82 anti-HBc (+) transplant patients, 68% of recipients achieved a robust immune response, thus not requiring antiviral therapy. Two patients (2.4%) developed DNHB infection, one of which was due to an escape mutant. With a median follow-up of 150 months, the overall 10-year patient and graft survival rates were significantly worse in recipients of anti-HBc (+) grafts than those of anti-HBc (-) grafts (85.2 vs 93.4%, *P*=0.026; 85.1 vs 93.4%, *P*=0.034, respectively). Additionally, the 10-year patient and graft outcomes of the anti-HBc (+) graft recipients were significantly worse than those of the anti-HBc (-) graft recipients after excluding early mortality and nongraft mortality values (90.8 vs 96.6%, *P*=0.036; 93.0 vs 98.3%, *P*=0.011, respectively).

**Conclusion:**

Our long-term follow-up study demonstrates that active vaccination is a simple, cost-effective strategy against DNHB infection in anti-HBc (+) graft patients, whereby the need for life-long antiviral therapy is removed. Notably, both the anti-HBc (+) grafts and patients exhibited inferior long-term survival rates, although the exact mechanisms remain unclear.

## Introduction

Highlights

- Anti-HBc (+) grafts can be a safe, effective, and practical way to address the problem of organ shortages.
- Active vaccination could remove the need for lifelong antiviral therapy, while the incidence of de novo hepatitis B infection and escape mutants are relatively rare.
- As long-term graft survival rates remain inferior to those from anti-HBc (-) donors.
- Avoiding the long-term use of antiviral therapy can be more cost-effective while eliminating the potential risk of teratogenicity in females of child-bearing age.

Liver transplantation (LT) is the most effective treatment for pediatric patients with end-stage liver disease, presenting excellent long-term survival rates^1,2^, although it has been limited by donor shortages. Thus, to expand the donor pool, many medical centers have amended their acceptance policies to include marginal organs that were previously rejected, including grafts from donors with HBsAg (-) but anti-HBc (+), particularly in regions with endemic HBV^3,4^. However, these grafts carry the risk of de novo hepatitis B infection (DNHB), with post-transplant infection rates ranging from ~10 to 25%, depending on the serological status of the liver graft recipients. The highest risk has been noted in hepatitis B (HBV) naïve recipients, with anti-HBs (-)/anti-HBc (-), as compared to recipients with anti-HBs (+)/anti-HBc (+)^5,6^. Additionally, a meta-analysis reported that HBV-naïve patients receiving anti-HBc (+) liver grafts had the highest risk of DNHB, up to 64% in patients without post-transplant HBV prophylactic therapy^7^.

While several strategies have been suggested to reduce DNHB infection, systemic reviews have shown similar effects among various types of post-transplant prophylaxes against DNHB^6,8^. However, many medical centers use the prophylaxis strategy indefinitely, thereby introducing issues of inconvenience and cost. Our previous investigation showed that an anti-HBs titer >1000 IU/l ensured the prevention of DNHB^4^. Therefore, our protocol recommends that recipients with a pretransplant anti-HBs titer >1000 IU/ml do not require antiviral therapy or hepatitis B immune globulin (HBIG), except for post-transplant HBV vaccine boosters^4,9,10^. Although, the long-term impacts of a pretransplant robust immune response after vaccination against DNHB remains unclear. Thus, the primary outcome of this study was to investigate the efficacy of a robust immune response over a 10-year follow-up period.

Despite reducing the incidence of DNHB to a satisfactory level with appropriate post-LT prophylaxis, the long-term patient and graft survival rates have shown conflicting results in non-HBV recipients with anti-HBc (+) grafts. More specifically, one study revealed similar 1-year, 3-year, and 5-year patient survival and graft survival rates, regardless of the presence of anti-HBc in the graft^11^. On the contrary, a separate investigation reported a reduction in the graft survival rate among non-HBV recipients with LT from anti-HBc (+) donors^12^; of note, the graft outcome was not directly associated with the occurrence of DNHB. In addition, the results of the long-term graft and survival outcomes were based on observations of adult LT recipients. Since investigations into the long-term graft outcomes from anti-HBc (+) donors in pediatric LT patients remains scarce, the secondary outcome here was to evaluate the long-term graft outcome and patient survival rates of anti-HBc (+) grafts in pediatric liver recipients.

## Material and methods

### Patient selection

This retrospective study conformed to the ethical guidelines of the Declaration of Helsinki and the work has been reported in line with the strengthening the reporting of cohort, cross-sectional, and case–control studies in surgery (STROCSS) criteria^13^. In this study, we employed active HBV vaccination combined with antiviral therapy as the prophylaxis against DNHB. Our previous study found that an anti-HBs titer >1000 IU/ml was effective at preventing the occurrence of DNHB^14^, thus removing the need for antiviral therapy in recipients with a robust response (anti-HBs titer >1000 IU/ml). Since 2002, antiviral therapy has only been used in recipients with a pretransplant nonrobust response (anti-HBs titer <1000 IU/l). The present study included 204 consecutive pediatric LDLT recipients enrolled at a tertiary medical center from February 2002 to February 2016, with follow-up to December 2023. The current study was approved by the Institutional Review Board (202001927B0). Donor variables were age, sex, relation to the recipient, anti-HBc positivity, and BMI. Recipient variables were age, sex, weight, underweight status, diagnosis of biliary atresia, Pediatric End-stage Liver Disease (PELD) score, HBV serostatus, number of HBV vaccine boosters, white blood cell count, hemoglobin, serum creatinine, platelet count, INR, alanine transaminase (ALT), Aspartate transaminase (AST), albumin, and total bilirubin level. Intraoperative and postoperative data were also included. The incidence of DNHB and the long-term graft and patient survivals were compared between recipients who received HBcAb positive and HBcAb negative grafts.

### Operative procedures and immunosuppression protocol

Surgical techniques and peri-operative care for the LDLT have been previously detailed^1,15^. All patients received a standard immunosuppressive therapy with cyclosporine, azathioprine, and prednisolone. Azathioprine was discontinued within 3 months, and prednisolone was gradually withdrawn 3 to 6 months post-transplant with no acute rejection episode^16^. In ABO incompatible recipients under 2 years of age, aside from shifting the backbone of immunosuppressants to tacrolimus, no additional pretransplant procedure nor medication were given.

### Prevention of DNHB

Prior to the transplant procedures, HBV vaccination with a single dose of recombinant HBV vaccine (20 μg, Engerix-B; Smithkline Beecham Biologicals) was given to the recipients, with repeated vaccinations administered to achieve an anti-HBs titer >1000 IU/l at the time of transplantation, providing that the recipient’s condition was stable. For patients who achieved a pretransplant anti-HBs titer >1000 IU/l, no post-transplant antiviral prophylaxis was given, aside from HBV booster vaccinations to maintain the anti-HBs titer >1000 IU/l after tapering off prednisolone. For patients with a low anti-HBs titer (<1000 IU/l) at the time of transplant, antiviral therapy, (lamivudine, GlaxoSmithKline Inc.) was given at a dose of 4 mg/kg/day for at least 2 years post-transplant, together with the HBV vaccinations after tapering corticosteroids. Antiviral therapy was discontinued with a post-transplant anti-HBs titer >1000 IU/ml; otherwise, the therapy was continued indefinitely. Of note, when it became available in 2008, lamivudine was replaced with entecavir (BARACLUDE, Patheon Inc.).

### Definition of outcomes

DNHB was defined as HBsAg seropositivity occurring in two consecutive tests. Once the recipient was determined to be positive of HBsAg, HBV DNA was extracted from the HBsAg sera, and was amplified by polymerase chain reaction followed by direct sequencing of the nucleotide sequences encoding the determinant of HBsAg (aa 116–160) using a dye terminator cycle sequencing quick start kit (Applied Biosystems). DNHB was treated with lamivudine (shifting to entecavir in 2008), and tenofovir disoproxil fumarate (Gilead Science Inc.) was added if a mutant strain was identified.

### Statistical analysis

The baseline patient characteristics are presented in the descriptive statistics of Table 1. The categorical variables underwent *χ* ^2^ test, and the continuous variables were tested with the Mann–Whitney *U* test. Data were labeled as significant with a *P*-value <0.05. Predictive factors underwent univariate and multivariate analyses using binary logistic regression. All statistical analyses were performed using IBM SPSS for version 22.

**Table 1:** Baseline characteristics of recipients who received HBcAb positive and HBcAb negative grafts.

	HBcAb(+) (*n*=82)	HBcAb(-) (*n*=122)	*P*
Age (years, min-max)	1.36 (0.37–17.09)	1.10 (0.45–17.53)	0.089
Sex: Male (%)	48 (58.5)	61 (50)	0.231
Diagnosis			
Biliary atresia	67 (81.7)	109 (89.3)	0.120
Body weight (kg)	9.4 (5.3–57.3)	8.8 (5.5–69.5)	0.063
Growth delay: weight	33 (40.2)	65 (53.3)	0.005
1st visit anti-HBs titer (IU/l)	98.4 (16.9–272.3)	119.0 (29.0–467.8)	0.091
PELD score	10.5 (−3.3–17.0)	13.0 (6.5–19.3)	0.007
Recipient anti-HBc			0.428
Positive	11 (13.4%)	12 (9.8%)	
Negative	71 (86.6%)	110 (90.2%)	
Booster dose(s) before transplantation			0.144
Present	77 (93.9%)	107 (87.7%)	
Absent	5 (6.1%)	15 (12.3%)	
Median (range )	3.7 (0–11)	2.7 (0–9)	0.006
Anti-HBs titer before transplant			
>1000	56 (68.3%)	65 (53.3%)	0.017
100–10 000	20 (24.4%)	35 (28.7%)	
<100	6 (7.3%)	22 (18.0%)	
WBC (×1000 u/l)	8.3 (5.0–11.7)	9.1 (6.0–12.1)	0.228
Hb (g/l)	9.8 (8.8–11.3)	9.9 (8.4–11.0)	0.628
Platelet (×1000 u/l)	16.7 (11.7–24.1)	16.7 (9.8–24.7)	0.562
INR	1.10 (0.99–1.26)	1.12 (1.05–1.39)	0.029
AST	214.5 (142.8–297.3)	235.0 (147.5–348.0)	0.287
ALT	133.0 (83.0–175.5)	142.5 (90.5–201.0)	0.327
Total bilirubin (g/dl)	9.8 (1.8–20.2)	15.9 (6.6–21.3)	0.018
Albumin (g/dl)	3.4 (2.8–3.8)	3.1 (2.7–3.7)	0.107

## Results

**Figure 1:**
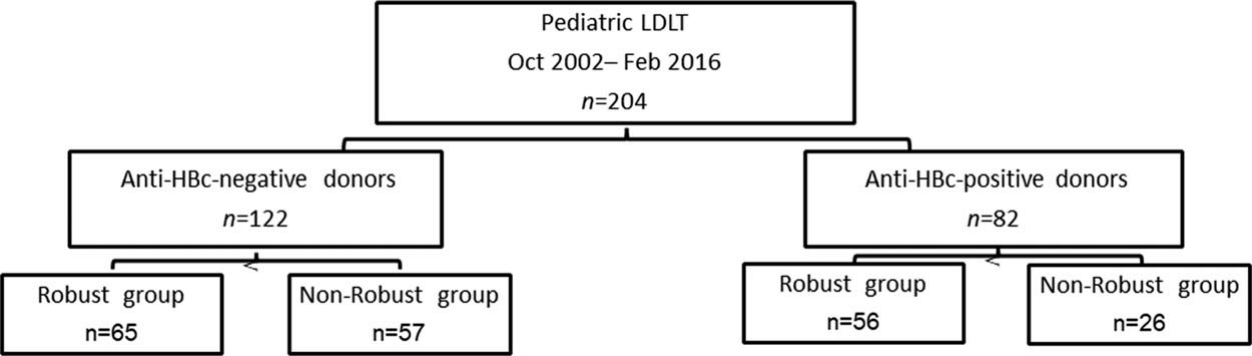
The flowchart of 204 pediatric LDLT recipients.

Among the study population, 82/204 (40.2%) recipients received anti-HBc (+) liver grafts, and 122 (59.8%) recipients received anti-HBc (-) liver grafts. After repeated pretransplant vaccinations, 65 recipients with anti-HBc (-) graft and 56 with anti-HBc (-) grafts achieved a robust immune response (Fig. 1).

### Demographics and pretransplant immune response

Regarding the baseline characteristics between the recipients receiving anti-HBc (+) and anti-HBc (-) grafts (Table 1), the variables including growth delay, PELD score, booster dose, pretransplant anti-HBs titer, international normalized ratio (INR), and total bilirubin level showed significant differences. Recipients with anti-HBc (-) grafts had a higher PELD score, INR, and higher total bilirubin levels. The recipients receiving anti-HBc (+) grafts were intentionally given more booster doses. All patients became positive for anti-HBs, while prior to transplant, 68.3% of recipients with ant-HBc (+) grafts and 53.3% of those with anti-HBc (-) grafts reached an anti-HBs titer >1000 IU/l. In the clinical data of the 204 recipients, the univariate and multivariate analyses identified no significant factors predictive of a robust response, including in the status of recipient anti-HBc, growth delay, and PELD score (Table 2).

**Table 2:** Univariate and multivariate analysis to predict pretransplant robust and nonrobust anti-HBs response.

	Univariate		Multivariate	
Variables	OR (95% CI)	*P*	OR (95% CI)	*P*
Age	1.045 (0.969–1.128)	0.254		
Sex	1.465 (0.833–2.576)	0.185		
Body weight (kg)	1.017 (0.989–1.045)	0.229		
Underweight	1.074 (0.614–1.878)	0.803		
Diagnosis (ba vs non ba)	1.276 (0.557–2.923)	0.565		
PELD score	0.978 (0.955–1.003)	0.079		
WBC (×1000 u/l)	1.016 (0.962–1.074)	0.566		
Hemoglobin (g/l)	1.132 (0.962–1.332)	0.135		
Platelet (×1000 u/l)	1.006 (0.982–1.030)	0.651		
INR	0.896 (0.605–1.326)	0.582		
ALT	0.998 (0.996–1.001)	0.138		
AST	0.998 (0.997–1.000)	0.088		
Total bilirubin(g/dl)	0.978 (0.953–1.003)	0.084		
Albumin(g/dl)	1.410 (0.920–2.162)	0.115		
No. of booster vaccination (before transplantation)	0.957 (0.841–1.089)	0.506		
Recipient anti-HBc	0.879 (0.366–2.111)	0.772		

(*n*=204).

### Maintenance of anti-HBs immunogenicity

Among the detailed long-term data of the 82 recipients having received anti-HBc grafts (Table 3), all of the robust responders had a pretransplant anti-HBs titer >1000 IU/l. In the nonrobust group, the anti-HBs titers were 100–1000 IU/l in 76.9% of recipients, and less than 100 IU/l in 23.1%. In the robust group, a majority of patients never received post-transplant antiviral treatment (96.4%); on the contrary, all patients in the nonrobust group received antiviral treatment against DNHB, of which 61.5% used it for over 2 years, with a median duration of 28.5 months. At the conclusion of the follow-up study period, all patients had discontinued use of antiviral agents, the anti-HBs titers maintained at >1000 IU/l in over 50% of recipients in both the robust and nonrobust responder groups. No significant differences in the acute cellular rejection rates and the general liver function tests were noted by the end of the follow-up period (Table 3).

**Table 3:** Sequential anti-HBs titers and clinical outcomes in anti-HBc(+) recipients.

	Total (*n*=82)	Robust (*n*=56)	Nonrobust (*n*=26)	*P*
Anti-HBs (before transplant)				
Median (IQR)	1600.0 (17.3–6941.0)	1600.0 (1600.0–1600.0)	254.5 (128.8–401.8)	*P*<0.001
>1000	56 (68.3%)	56 (100%)	0 (0%)	
100–1000	20 (24.4%)	0 (0%)	20 (76.9%)	*P*<0.001
<100	6 (7.3%)	0 (0%)	6 (23.1%)	
Lamivudine use				
Present	28 (34.1%)	2 (3.6%)	26 (100.0%)	*P*<0.001
Absent	54 (65.9%)	54 (96.4%)	0 (0%)	
Lamivudine duration				
≥24 month	16 (19.3%)	0 (0%)	16 (61.5%)	
<24 month	11 (13.4%)	2 (3.6%)	9 (34.6%)	*P*<0.076
No drug	55 (67.1%)	54 (96.4%)	1 (3.8%)	
Post-transplant vaccination				
Present	48 (58.5%)	30 (53.6)	18 (69.2)	*P*=0.180
Absent	34 (41.5%)	26 (46.4)	8 (30.8)	
Median (range )	2 (0–13)	1 (0–13)	2 (0–13)	*P*=0.114
Anti-HBs in the end of follow-up				
Median (or mean)	1064.0 (5.0–2133.0)	1266.0 (10.0–2000.0)	1039.5 (5.0–2133.0)	*P*=0.813
>1000	47 (57.3%)	33 (58.9)	14 (53.8)	
100–10 000	30 (36.6%)	20 (35.7)	10 (38.5)	*P*=0.632
<100	5 (6.1%)	3 (5.4)	2 (7.7)	
Liver function tests	(follow-up)			
ALT (median, min-max)	25.0 (6.0–378.0)	25.0 (6.0–378.0)	31.5 (10.0–142.0)	*P*=0.252
AST	33.5 (11.0–2483.0)	29.0 (11.0–2483.0)	40.5 (17.0–356.0)	*P*=0.100
Total bilirubin (g/dl)	0.9 (0.2–50.5)	0.8 (0.2–50.5)	1.1 (0.4–49.9)	*P*=0.153
Albumin (g/dl)	4.4 (2.3–5.2)	4.4 (2.3–5.2)	4.5 (3.4–4.9)	*P*=0.865
INR	1.1 (0.17–2.30)	1.1 (0.9–2.3)	1.1 (0.2–2.3)	*P*=0.701

### De novo HBV and escape mutant

**Figure 2:**
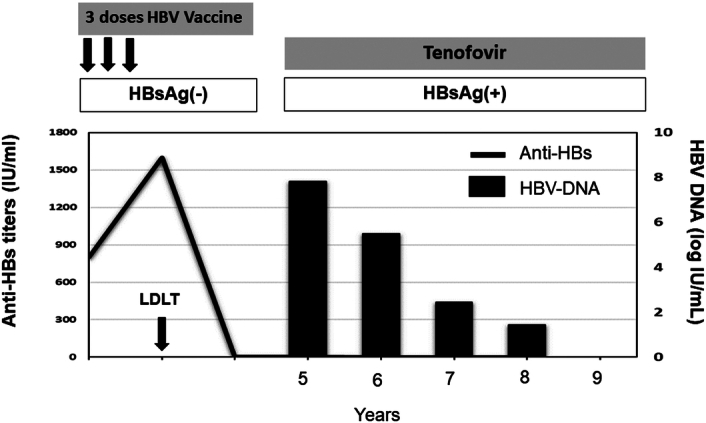
The series levels of the anti-HBs and HBV DNA and antiviral therapy in the patient developing escape mutant de novo HBV infection.

**Figure 3:**
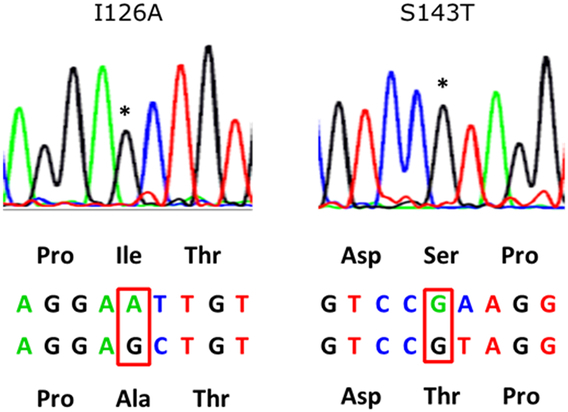
Two nucleotide mutants for the HBV surface gene were identified by sequence analysis in (Ile 126 Ala) and (Ser 143 Thr).

Two (2.4%) patients developed DNHB, both of whom were in the robust responder group. One patient contracted DNHB due to an escape mutant. This recipient (LDLT No. 743) was with biliary atresia and underwent transplantation at the age of 10 months. The pretransplant anti-HBc test was negative, and the anti-HBs titer increased to 1600 IU/l from 800 IU/l after 3 vaccinations. Additionally, the HBsAg test was positive at 60 months after LDLT, hence tenofovir disoproxil fumarate was administered (Fig. 2). Sequencing analysis for the HBV surface gene identified two nucleotide mutants at Ile126Ala (I126A) and Ser143Thr (S143T) (Fig. 3), compared with the HBV sequence provided by GenBank (KX067076). The liver function tests were within normal range and no cirrhotic change was noted on ultrasonography, while the HBV DNA was negative at 48 months after initiating antiviral therapy. The other case (LDLT1263) developed DNHB at 53 months after LT, with the DNA sequencing showing no mutant strain. After receiving anti-HBV therapy, the patient was negative for HBV DNA, with stable liver function.

### Patient and graft survival rates

We further analyzed the risk factors potentially influencing graft survival. The univariate analysis demonstrated that biliary atresia (HR 4.413, 95% CI: 1.566–12.435, *P*=0.005), anti-HBc (+) (HR 3.672, 95% CI: 1.162–11.602, *P*=0.027), Clavien–Dindo complication grade (HR 4.085, 95% CI: 1.764–9.458, *P*=0.001), and other specific surgical complications such as hepatic artery, biliary or portal vein events were unfavorable factors (Table 4). Additionally, the multivariate analysis revealed biliary atresia (HR 4. 070, 95% CI: 1.350–12.275, *P*=0.013), anti-HBc (+) (HR 3.323, 95% CI: 1.003–11.006, *P*=0.049) and HA complication (HR 18.079 95% CI: 6.336–51.58, *P*=0.001) as unfavorable factors.

**Table 4:** The risk factors influencing graft survival in pediatric LDLT recipients (*n*=204).

	Univariate		Multivariate	
Variables	HR (95% CI)	*P*	HR (95% CI)	*P*
Donor				
Age	1.00 (0.97–1.02)	0.898		
Sex	0.77 (0.26–2.25)	0.631		
BMI	1.04 (0.90–1.21)	0.586		
Age	0.96 (0.82–1.12)	0.596		
Sex	1.04 (0.38–2.87)	0.940		
Body weight (kg)	0.97 (0.91–1.04)	0.420		
Underweight	0.72 (0.26–2.04)	0.541		
Diagnosis (non ba vs ba)	4.41 (1.57–12.44)	0.005	4.07 (1.35–12.28)	0.013
PELD score	1.01 (0.97–1.05)	0.705		
Donor HBcAb (+ vs -)	3.67 (1.16–11.60)	0.027	3.32 (1.00–11.01)	0.049
Recipient HBcAb (+ vs -)	1.76 (0.49–6.25)	0.383		
ROBUST	1.19 (0.40–3.49)	0.757		
Booster dose(s) before transplantation				
YES/NO	0.75 (0.17–3.35)	0.710		
number	0.97 (0.77–1.24)	0.834		
Anti-HBs titer before transplant	1.00 (1.00–1.00)	0.787		
Postoperative				
Clavien–dindo (≥ 3 vs <3)	4.04 (1.46–11.15)	0.007		
Complication				
HA	12.77 (4.60–35.44)	0.001	18.08 (6.34–51.58)	0.001
BD	4.80 (1.35–17.03)	0.015		
Portal vein	3.31 (1.13–9.74)	0.029		
Immunosuppression				
Cyclosporine vs others	0.59 (0.19–1.85)	0.386		
Hospital stay	0.99 (0.97–1.02)	0.581		

Among our study cohort, there were 27 recipients who died during the study period, underwent retransplant or were on the waiting list, with surgical mortality, or a combination of two factors, as detailed in Table 5. In the anti-HBc (+) graft group, there were 18 graft events, including six early (<6 months) and nine late (>6 months) mortalities, and three retransplant cases who are still alive. In the anti-HBc (-) graft group, there were nine graft events, including four early and four late mortalities, and one retransplant case who is still alive.

**Table 5:** Causes of mortality/graft failure and indications for retransplantation.

Graft /Recipient anti-HBc	Retransplant (anti-HBc)/ Mortality (months)	LDLT No.	Early/Late	Cause	Graft-related
+/-	Negative/Mortality (1.9)	230	Early	Sepsis	No
+/-	Negative/Mortality (1.5)	291	Early	Hepatic artery thrombosis	Yes
+/-	Negative/Mortality (0.7)	299	Early	Lung hemorrhage, TTP	No
+/-	Negative/Mortality (0.7)	598	Early	PV thrombosis	Yes
+/-	Negative/Mortality (0.9)	1114	Early	Sepsis	No
+/-	Negative/Mortality (8.7)	151	Late	PTLD	No
+/+	Positive/Mortality (12.7)	390	Late	Sepsis	No
+/-	Negative/Mortality (16.3)	957	Late	PTLD	No
+/-	Negative/Mortality (33.3)	1157	Late	PTLD	No
+/-	Negative/Retransplant(104.7)/Mortality (113.1)	168	Late	Chronic rejection	Yes
+/+	Positive/Mortality (86.0)	447	Late	Drug incompliance	Yes
+/-	Negative/Mortality (131.6)	171	Late	Unknown graft failure	Yes
+/-	Negative/Mortality (171.7)	181	Late	Unknown graft failure	Yes
+/-	Negative/Mortality (95.4)	297	Late	Biliary cirrhosis	Yes
+/-	Negative/Retransplant (0.53)	287	Early	HA thrombosis	Yes
+/-	Negative/Retransplant (154.1)	144	Late	Liver A-V malformation	Yes
+/-	Negative/Retransplant (116.8)	487	Late	Unknown graft failure	Yes
+/-	Negative/On Retransplant list (226)	211	Late	Unknown graft failure	Yes
-/-	Negative/Mortality (0.8)	233	Early	PV thrombosis	Yes
-/-	Negative/Mortality (3.35)	385	Early	Sepsis	No
-/-	Negative/Morality (1.08)	465	Early	Intracranial hemorrhage	No
-/+	Positive/Retransplant (0.4)/Mortality (0.4)	1060	Early	HA thrombosis	Yes
-/-	Negative/Mortality (18.0)	335	Late	Sepsis	No
-/-	Negative/Mortality (12.7)	442	Late	Sepsis	No
-/-	Negative/Mortality (39.4)	1218	Late	Sepsis	No
-/+	Positive/Retransplant (6.1)/Mortality(18.0)	123	Late	Biliary cirrhosis/ Veno-occlusive disease	Yes
-/-	Negative/Retransplant (0.2)	735	early	HA thrombosis	Yes

**Figure 4:**
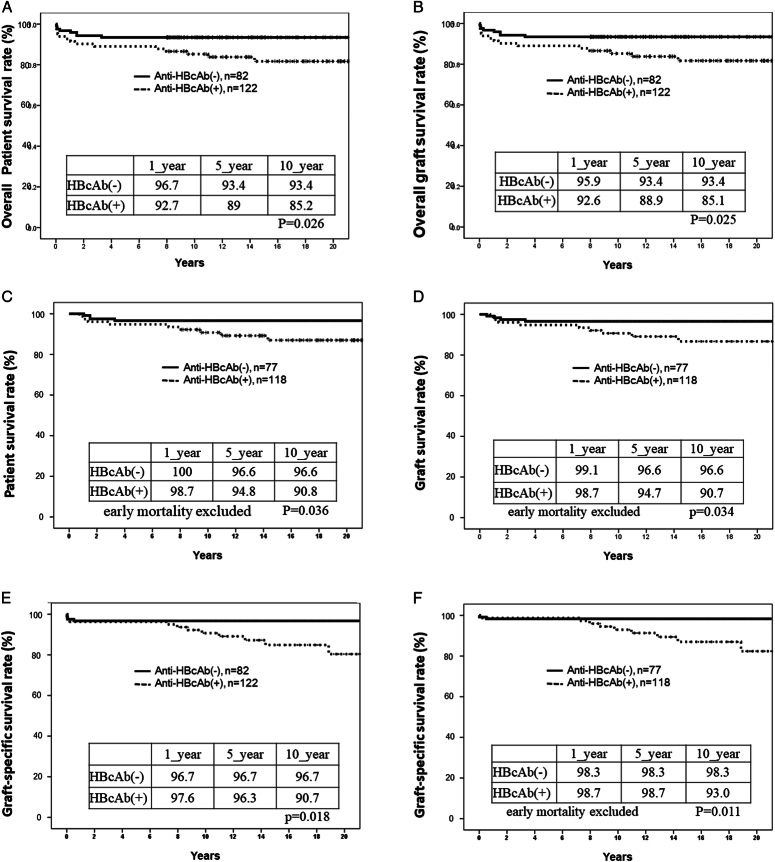
Comparisons of long-term survival rates with anti-HBc (+) grafts and anti-HBc (-) grafts. (A) Overall patient survival rates in the two groups. (B) Overall graft survival rates in the two groups. (C) Patient survival rates without early mortality in the two groups. (D) Graft survival rates without early mortality in the two groups. (E) Graft-specific survival rates in the two groups. (F) Graft-specific survival rates without early mortality in the two groups.

The median overall follow-up was 150 (0.3–255.5) months. The 1-year, 5-year, and 10-year overall patient survival rates of the recipients with anti-HBc (+) grafts were 92.7, 89.0, and 85.2%, respectively, significantly inferior to those with anti-HBc (-) grafts, which were 96.7, 93.4, and 93.4%, respectively (*P*=0.026) (Fig. 4A).

Taking into account all mortalities for graft failure, the 1-year, 5-year, and 10-year graft survival rates of the recipients with anti-HBc (+) grafts were 92.6, 88.9, and 85.1%, respectively, which were slightly worse than those with anti-HBc (-) grafts at 95.9, 93.4, and 93.4%, respectively (*P*=0.025) (Fig. 4B).

To examine the impact of graft anti-HBc status on long-term survival, the early mortalities (<6 months after LDLT), mostly surgery-related, were removed from the analysis. Again, the 10-year patient survival rate of the anti-HBc (+) grafts were significantly inferior to those with anti-HBc (-) grafts (90.8 vs 96.6%, *P*=0.036; Fig. 4C). Similarly, the 10-year graft survival rate of the anti-HBc (+) grafts, excluding early mortalities, was also significantly inferior to those with anti-HBc (-) grafts (96.6 vs 90.7%, *P*=0.034; Fig. 4D).

We further investigated the impact of anti-HBc (+) status on graft survival. For the statistical analysis, causes of morality from sepsis, PTLD, and pulmonary/cranial hemorrhage were considered of nongraft origin, while thrombosis, biliary cirrhosis, and retransplant were considered as graft survival (graft-specific survival). The analysis revealed that 11 of 18 graft events in the anti-HBc (+) graft group and 4 of 9 graft events in the anti-HBc (-) graft group were related to graft origin (Table 5). In addition, the overall 10-year graft-specific survival rate in the anti-HBc (+) was significantly inferior to that of the anti-HBc (-) group (90.7 vs 96.7%, *P*=0.018; Fig. 4E). The result was similar when early mortalities were excluded (93.0 vs 98.3%, *P*=0.011; Fig. 4F).

## Discussion

LT for pediatric patients with end-stage liver disease provides excellent long-term outcomes. However, organ shortage remains a critical issue, particularly in Asia. In areas where HBV has been an epidemic disease, such as many east Asian countries, it is common to use anti-HBc (+) grafts. The present study provides valuable insights into the impact of anti-HBc (+) grafts on the incidence of DNHB under a well-established managing protocol with long-term follow-up.

There is currently no consensus regarding the post-transplant protective titer value of anti-HBs for preventing DNHB. A plausible explanation for recipients developing DNHB, albeit in the presence of anti-HBs, may be that the titer value is insufficient to prevent occurrence. In the normal population, a postvaccination anti-HBs titer >10 IU/l is considered sufficient to be protective. Meanwhile, to prevent DNHB, it is recommended that post-LT recipients with anti-HBc (+) grafts achieve an anti-HBs titer >500 IU/l by administration of HBIG^17^. One study reported that an anti-HBs titer >200 IU/l by vaccination significantly reduced the incidence of DNH to 4.3% in pediatric recipients receiving anti-HBc (+) grafts^18^. Notably, our previous study showed that an anti-HBs titer >1000 IU/l after vaccination significantly reduced the incidence of DNHB from 15.4 to 0% in pediatric patients^19^. In theory, the higher the anti-HBs titer value, the more protection can be provided, whereby a higher anti-HBs titer value would neutralize the potential viral antigen coming from the anti-HBc (+) grafts^20,21^.

Poor nutrition and advanced cirrhosis may hinder the response of the vaccine^22^, although we did not identify any predictive factors related to the generation of anti-HBs after repeated vaccinations. A majority of our patients were positive for anti-HBs, with titers >1000 IU/l in nearly 60% of recipients, as the primary diagnosis of pediatric patients was cholestatic biliary atresia. Furthermore, active vaccination may raise concerns regarding escape mutants of a determinant within HBsAg^23^. In this series, just one patient (~1.2%) developed an escape mutant. Of note, the patient recovered after antiviral therapy. Thus, we believe that the benefits of our current policy recommending prophylaxis for DNHB outweigh the potential risks.

With the development of a new generation of drugs, antiviral monotherapy is a simple choice to prevent the occurrence of DNHB in most medical centers^6^. However, when pediatric recipients eventually reach child-bearing age, antiviral therapy will raise concerns of teratogenicity (mainly category B)^24^. In addition, there is a relatively good chance of generating sufficient anti-HBs titers in pediatric recipients. Considering these two factors, avoiding the long-term use of antiviral therapy can be more cost-effective while eliminating the potential risk of teratogenicity in females of child-bearing age.

Studies have reported conflicting results in terms of graft and patient survival when using anti-HBc (+) grafts in recipients without serological evidence of HBV infection^25,26^. Using anti-HBc (+) grafts may not result in inferior graft survival rates in adult recipients, particularly in HBV recipients^27^. However, evidence suggests a negative impact on outcome for non-HBV recipients with anti-HBc (+) grafts^23,28^. In our study, anti-HBc (+) grafts led to a poorer outcome during the 10-year follow-up in pediatric recipients, most of whom were non-HBV patients. The graft survival of rate of anti-HBc (+) grafts was significantly worse than that of anti-HBc (-) grafts, both by unadjusted comparison or after adjustment, although DNHB infection was well-controlled under our protocol or by those of other published strategies. It must be noted that the underlying mechanisms require further investigation. Among the anti-HBc (+) grafts, occult HBV infection may be present; however, we did not routinely check the HBV viral load in our anti-HBc (+) donors. Meanwhile, the prevalence of occult HBV infection may vary, although it is indeed more common in endemic regions^20,29^. Hepatitis B virus causes liver damage by integrating its DNA into the host genome, producing proteins with transforming capabilities, and the subsequent introduction of necroinflammation^21,30–32^. Furthermore, a history of pre-existing HBV infection may cause the subclinical hepatocyte damage that could be aggravated by post-transplant insults such as ischemia-reperfusion injuries and acute cellular rejection. Accordingly, pre-existing suboptimal graft quality and post-LT insults may explain, at least in part, the inferior survival rate we found for the anti-HBc (+) grafts.

A particular strength of the present study is in the inclusion of a consecutive cohort of pediatric LDLT recipients with long-term details of follow-up, although there are several limitations. First, this was a retrospective study conducted at a single medical center. Second, the HBV prophylaxis changed from lamivudine to entecavir in 2008, therefore we were unable to evaluate the risk of developing lamivudine resistance. In addition, the DNA level data for the donor and graft tissues were lacking and this study was conducted in an area with a high prevalence of anti-HBc positivity. Lastly, the study followed the institutionalized protocol without scientifically control group from other centers for external validation.

## Conclusion

Our study demonstrates that using anti-HBc (+) grafts can be a safe, effective and practical way to address the problem of organ shortages. Active vaccination could remove the need for lifelong antiviral therapy, while the incidence of DNHB and escape mutants are relatively rare. As long-term graft survival rates remain inferior to those from anti-HBc (-) donors, the avoidance of any further parenchymal damage from any kind of insult in these particular grafts is advised.

## Ethical approval

The current study was approved by the Chang Gung Medical Foundation Institutional Review Board (202001927B0).

## Consent

Identifying details have been omitted.

## Source of funding

This work was supported by grants from health and welfare surcharge of tobacco products, Ministry of Health and Welfare, Taiwan (MOHW111-TDU-B-221-114009, and MOHW112-TDU-B-222-124009 to Chen CL and from Kaohsiung Chang Gung Memorial Hospital, Taiwan (CMRPG8L0331, CMRPG8L0811 and CMRPG8M0371 to Lin CC).

## Author contribution

C.-C.Y.: design study and draft manuscript; Y.-H.L.: interpretation of data and revise the work; W.Z.E.: data collection and draft manuscript; I.-H.C.: data analysis and draft manuscript; S.-H.W.: perform study and acquisition of data; Y.-C.C.: perform study and acquisition of data; C.-L.C.: conception of the work; C.-C.L.: design study and editing manuscript.

## Conflicts of interest disclosure

The authors of this manuscript have no conflicts of interest to disclose.

## Research registration unique identifying number (UIN)

This retrospective study conformed to the ethical guidelines of the Declaration of Helsinki and the registration no was ClinicalTrials.gov ID: NCT06290726.

## Guarantor

Chih-Che Lin is the guarantor.

## Data availability statement

Raw data were generated at Kaohsiung Chang Gung Memorial Hospital. Data supporting the findings of this study are available from the corresponding author C.C. Lin upon request.

## Provenance and peer review

Not commissioned, externally peer-reviewed.
